# Small bowel perforation and peritonitis: a rare life-threatening complication of extracorporeal shock wave lithotripsy (case report)

**DOI:** 10.11604/pamj.2025.52.34.49223

**Published:** 2025-09-22

**Authors:** Hossameldin Alnawasra, Faisal Ahmed, Munzir Obaid, Maged Alghashmi, Siddig Abdallah, Nizar Bouchiba, Morshed Salah

**Affiliations:** 1 Urology Section, Hazm Mebaireek General Hospital, Hamad Medical Corporation, Doha, Qatar,; 2 Department of Urology, School of Medicine, Ibb University, Ibb, Yemen,; 3 Acute Care Surgery, Hazm Mebaireek General Hospital, Hamad Medical Corporation, Doha, Qatar,; 4 College of Medicine, Qatar University, Doha, Qatar

**Keywords:** ESWL, bowel perforation, peritonitis, surgical emergency, case report

## Abstract

Extracorporeal shock wave lithotripsy (ESWL) is a cornerstone non-invasive therapy for urolithiasis. While major complications are rare, bowel perforation represents a serious life-threatening event. We present the case of a 36-year-old male with a history of recurrent renal stones who developed an acute abdomen four hours after supine ESWL for a right proximal ureteral calculus. Computed tomography revealed pneumoperitoneum and mesenteric stranding. Emergency laparotomy confirmed a jejunal perforation with secondary peritonitis, which was managed with primary repair and ureteral stenting. The patient made a full recovery. This case highlights a severe but rare complication of a common urological procedure. It underscores the critical need for high clinical suspicion in any patient presenting with abdominal pain post-ESWL, as prompt diagnosis via cross-sectional imaging and immediate surgical intervention are paramount for survival. A multidisciplinary approach is essential for optimal management.

## Introduction

Extracorporeal shock wave lithotripsy (ESWL), first introduced in 1980, remains a cornerstone non-invasive treatment for upper urinary tract calculi ≤20 mm in diameter [1]. The procedure utilizes high-intensity acoustic pulses to fragment stones, facilitating their spontaneous passage while minimizing damage to surrounding tissues. ESWL is generally safe, with major complications reported in less than 5% of cases. However, rare but serious adverse events, including gastrointestinal perforation, can occur. This risk is thought to be higher in patients with a history of abdominal surgery due to possible adhesions that may fix bowel loops within the path of shockwaves [2-4]. The early recognition and management of such complications are critical to prevent life-threatening conditions like peritonitis [2,3,5]. We present a case of small bowel perforation following ESWL in a 36-year-old male with no prior abdominal surgery, highlighting that this severe complication can occur even in the absence of classic risk factors. This case underscores the imperative for heightened vigilance and prompt intervention in any patient presenting with abdominal pain after ESWL.

## Patient and observation

**Figure 1:**
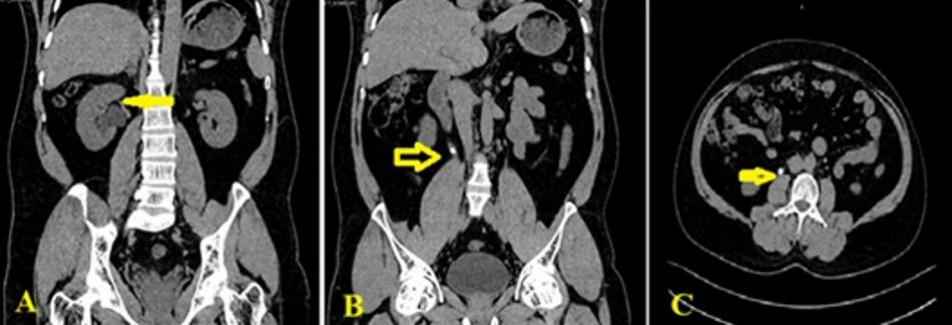
pre-procedural imaging of right ureteral obstruction: A) non-contrast axial Computed Tomography (CT) image of the abdomen demonstrating moderate hydronephrosis (arrow) secondary to an impacted ureteral calculus; B) axial CT image showing an 8 x 5mm hyperdense stone (arrow) at the right proximal ureter, adjacent to the lower border of the L3 vertebra; C) coronal reformatted CT image confirming the stone's location (arrow) with associated proximal ureteral dilation

**Patient information:** a 36-year-old male presented with sudden onset of severe abdominal pain four hours after undergoing extracorporeal shock wave lithotripsy (ESWL) for a right proximal ureteral calculus (Figure 1). The pain was initially localized to the periumbilical region, radiating to the right flank, and rapidly progressed to become generalized and severe. Associated symptoms included nausea, vomiting, obstipation, hematuria, and dysuria. The patient had a notable history of recurrent nephrolithiasis managed with multiple prior ESWL treatments. He denied any prior abdominal surgeries, chronic illnesses, or regular medication use.

**Clinical findings:** on presentation, the patient was afebrile and hemodynamically stable. Abdominal examination revealed marked distension and tenderness predominantly in the periumbilical region. No guarding or rebound tenderness was evident.

**Figure 2:**
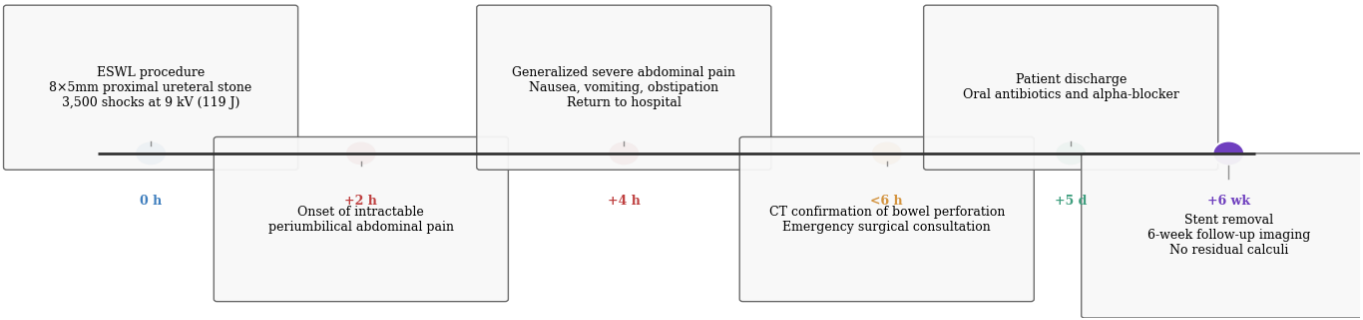
timeline of the clinical course following ESWL: chronological representation of key events from the initial procedure to follow-up, highlighting the rapid onset of symptoms, diagnostic steps, surgical intervention, and patient recovery

**Timeline of current episode:** a chronological summary of key clinical events following ESWL is illustrated in Figure 2.

**Figure 3:**
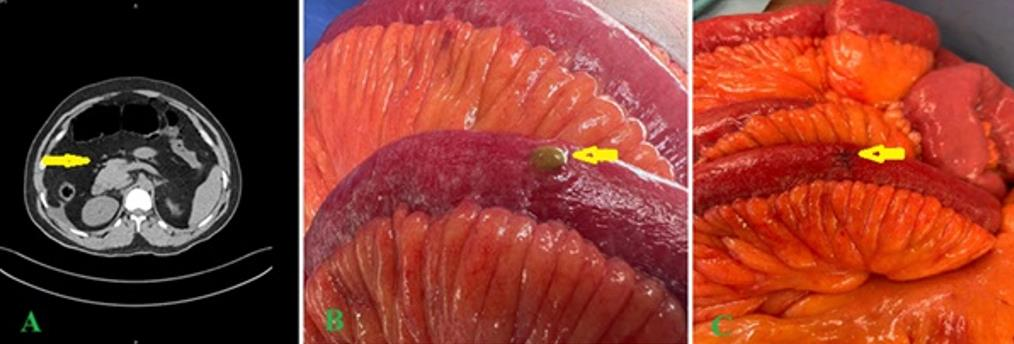
radiologic and intraoperative findings of small bowel perforation following extracorporeal shock wave lithotripsy (ESWL): A) contrast-enhanced axial CT image of the abdomen demonstrating signs of acute hollow viscus perforation, including pneumoperitoneum (free intraperitoneal air, arrow), perihepatic free fluid (asterisk), and mesenteric fat stranding (arrowhead); B) intraoperative photograph revealing a 2mm perforation (arrow) in the jejunum, approximately 120cm distal to the ligament of Treitz; C) intraoperative photograph showing the completed primary repair of the enterotomy (arrow) with interrupted absorbable sutures

**Diagnostic assessment:** laboratory investigations revealed leukocytosis (13x10^3^/µL) with neutrophilia (80%), while renal and hepatic functions remained within normal limits. Abdominal radiographs revealed dilated bowel loops with faint radiopacities noted in the right distal ureter. Contrast-enhanced computed tomography (CT) of the abdomen and pelvis demonstrated hallmark features of intestinal perforation: axial images revealed pneumoperitoneum (free intraperitoneal air, arrow), perihepatic free fluid, and prominent mesenteric fat stranding (Figure 3A). Additional free fluid was noted in the right paracolic gutter. Coronal CT images identified a column of stone fragments (steinstrasse) at the ureterovesical junction, accounting for obstructive uropathy (Figure 3B). These imaging findings confirmed jejunal perforation secondary to ESWL, guiding prompt surgical intervention and ureteral stenting.

**Diagnosis:** secondary peritonitis due to jejunal perforation was diagnosed as a complication following ESWL. Concurrent obstructive uropathy due to steinstrasse formation at the ureterovesical junction was also established.

**Therapeutic interventions:** emergency diagnostic laparoscopy was initiated but converted to open laparotomy due to dense adhesions limiting operative exposure. A 2mm jejunal perforation was identified 120cm distal to the ligament of Treitz and repaired primarily with 3-0 absorbable sutures (Figure 3C). Thorough peritoneal lavage was performed. Concurrent cystoscopy and retrograde pyelography facilitated placement of a 4.8 Fr magnetic double-J ureteral stent (UroTech®) to alleviate ureteral obstruction.

**Follow-up and outcome of interventions:** postoperative recovery was uneventful. The patient was discharged on postoperative day five with oral antibiotics and an alpha-blocker to aid stone fragment passage. At six-week follow-up, kidney-ureter-bladder X-ray confirmed correct stent positioning and clearance of residual calculi. The stent was removed cystoscopically without complications, and the patient made a full recovery.

**Patient perspective:** “The pain after the procedure was sudden and far worse than any stone pain I've experienced before. I immediately knew something was wrong. I am grateful for the rapid medical response and successful surgery despite the frightening experience.”

**Informed consent:** written informed consent was obtained from the patient for publication of this case report and associated images.

## Discussion

This case highlights bowel perforation as a rare but serious complication of ESWL, underscoring the delicate balance between efficacy and safety in this gold-standard treatment for urinary calculi. While ESWL boasts a favorable safety profile overall, our experience reinforces that its success hinges on meticulous patient selection and technique optimization, particularly when addressing shockwave-resistant stones (e.g., cystine or dense calcium compositions), high stone burden, or anatomically challenging locations such as narrow calyceal systems [3]. These factors, compounded by chronic infections or prior interventions, may predispose patients to complications ranging from obstructive fragments to the bowel injury we encountered.

The technical parameters of our case align with several high-risk features identified in historical perforation events. Treatment in the supine position, observed in 75% of prior cases [2,6,7], likely exacerbated bowel exposure to shockwave energy. Our protocol-delivering 3,500 shocks at 9 kV (total 119 J)-exceeded the 6-7 kV range typical of most perforation reports [3] but remained within documented extremes. Notably, perforations have occurred even at &#8804 6.5 kV (4 of 16 cases in one review) [3], suggesting a non-linear dose-response relationship where cavitation effects may peak at 7-8 kV before shielding attenuates tissue damage at higher energies. This phenomenon mirrors in vitro studies of the Dornier HM3, where bubble clouds generated at intermediate energies (18-20 kV) amplified secondary collapse pressures, whereas excessive energies (>24 kV) paradoxically reduced efficacy through energy dissipation [8].

The Dornier HM3's legacy as a high-pressure (40-50 MPa), narrow-focus lithotripter is particularly relevant here. Its sharply concentrated energy profile - contrasting with broader-focus devices like the XX-Es-intensifies cavitation and shear stresses at the target, but also increases off-target risks in anatomically vulnerable regions [8]. Our use of a third-generation Dornier Delta III pro (EMSE 180) demonstrates that while modern systems improve targeting precision, they cannot fully eliminate perforation risks when other factors converge. This is especially true for proximal ureteral stones at L3, where the proximity of bowel loops and shockwave scattering in confined spaces may create secondary pressure peaks up to 4 cm beyond the focus-a effect well-documented with HM3-style focusing [8]. The Dornier HM3's narrow focal zone (40-50 MPa) intensifies cavitation, as demonstrated in computational models by Krimmel *et al*. [8], but modern systems like the Delta III mitigate this through broader focusing [9]. Nevertheless, as shown by Fontanet *et al*., supine positioning and energies >7 kV persist as independent risk factors [3].

Most ESWL complications are transient (e.g., hematuria in 85-100%, colic in 30-50% [3,4], but our case exemplifies the <0.1% risk of life-threatening bowel injury [2-4,10]. The pathophysiology involves direct shockwave trauma and cavitation-induced microjetting, particularly in patients with adhesions from prior surgery. Our patient's rapid symptom onset (4 hours post-ESWL)-versus the 5-day delay reported by Huang *et al*. [10]-highlights the variability in perforation presentations. The diagnostic triad of pneumoperitoneum, mesenteric stranding, and neutrophilic leukocytosis on CT proved pivotal, as did our adherence to trauma principles: laparoscopic exploration (converted due to adhesions) with simultaneous ureteral stenting.

This experience prompts critical reflections on technique, including energy modulation, where lower kV protocols (e.g., starting at 6-7 kV with gradual titration) may mitigate visceral injury without compromising stone fragmentation; positioning alternatives, as the transgluteal approach for proximal ureteral stones could reduce bowel exposure, although data are limited; and enhanced monitoring, as real-time ultrasound coupling or cavitation detectionâ-features of third-generation systems like the Delta III's Optivision-may help balance efficacy and safety. Preventive strategies must emphasize preprocedural risk stratification (e.g., assessing surgical history, body habitus) and explicit informed consent regarding rare complications [8]. Future efforts should refine risk prediction models and shockwave delivery systems to minimize off-target effects, building on insights from HM3-era cavitation studies [8].

This case prompts consideration of several safety strategies: initiating lithotripsy at lower energy settings with gradual escalation, evaluating alternative patient positions such as the transgluteal approach to reduce bowel exposure, and using real-time ultrasound coupling to optimize shockwave delivery. Comprehensive pre-procedural risk stratification, including evaluation of surgical history and individual anatomy, alongside informed consent addressing rare but serious risks, should become standard practice-particularly in resource-limited settings.

## Conclusion

This case demonstrates that bowel perforation, though rare, is a devastating complication of ESWL that can occur even in the absence of classic risk factors like previous abdominal surgery. The supine position and specific energy parameters are significant technical considerations that require careful attention. This report underscores the critical importance of a high index of suspicion for any patient presenting with acute abdominal pain following ESWL. Prompt diagnosis with computed tomography and immediate multidisciplinary management involving urology and general surgery are essential to prevent mortality. Ultimately, thorough preoperative counseling and meticulous technique remain the best defenses against this unforeseen complication.
